# Effects of a 15-month anti-TNF-α treatment on plasma levels of glycosaminoglycans in women with rheumatoid arthritis

**DOI:** 10.1186/s13075-018-1711-z

**Published:** 2018-09-18

**Authors:** Anna Szeremeta, Agnieszka Jura-Półtorak, Ewa Maria Koźma, Andrzej Głowacki, Eugeniusz Józef Kucharz, Magdalena Kopeć-Mędrek, Krystyna Olczyk

**Affiliations:** 1 Department of Clinical Chemistry and Laboratory Diagnostics, School of Pharmacy with the Division of Laboratory Medicine in Sosnowiec, Medical University of Silesia in Katowice, Jedności 8, 41-200 Sosnowiec, Poland; 2 Department of Internal Medicine and Rheumatology, School of Medicine in Katowice, Medical University of Silesia in Katowice, Ziołowa 45/47, 40-635 Katowice, Poland

**Keywords:** Rheumatoid arthritis, Tumor necrosis factor-alpha inhibitors, Glycosaminoglycans, Keratan sulfate, Hyaluronic acid

## Abstract

**Background:**

In this study, the effect of 15-month anti-tumor necrosis factor alpha (TNF-α) treatment on circulating levels of plasma sulfated glycosaminoglycans (GAGs) and the nonsulfated GAG hyaluronic acid (HA) in female rheumatoid arthritis (RA) patients was assessed.

**Methods:**

Plasma was obtained from healthy subjects and RA women treated with TNF-α antagonists (etanercept or adalimumab or certolizumab pegol) in combination with methotrexate. GAGs were isolated from plasma samples using ion exchange low-pressure liquid chromatography. Total sulfated GAGs were quantified using a hexuronic acid assay. Plasma levels of keratan sulfate (KS) and HA were measured using immunoassay kits.

**Results:**

Total sulfated GAGs and HA levels were higher in female RA patients before treatment in comparison to healthy subjects. KS levels did not differ between RA women and controls. Anti-TNF-α treatment resulted in normalization of plasma total GAG and HA levels in RA patients, without any effect on KS levels.

**Conclusions:**

Our results suggest that anti-TNF-α therapy has a beneficial effect on extracellular matrix remodeling in the course of RA.

## Background

Rheumatoid arthritis (RA) is a chronic, systemic, autoimmune connective tissue disease characterized by nonspecific arthritis of symmetric joints, as well as progressive articular cartilage degradation and bone erosion [1]. In the course of the disease there are numerous extra-articular organ manifestations which are the major causes of fast-growing patient disability. RA is associated with a higher mortality rate compared to that of the general population. RA affects approximately 0.5–1.5% of the world’s population at any age. The incidence peaks in the 40–50 age group and women suffer from RA nearly two or three times more frequently than men [2–4].

Despite many studies, the causes of RA are still not completely known [3–5]. Tumor necrosis factor alpha (TNF-α) is one of the main proinflammatory cytokines, playing a key role in the pathogenesis of the disease. It has been shown that TNF-α stimulates catabolic processes in the cartilage tissue and periarticular structures and significantly affects the induction and persistence of inflammation [4–7]. Some of the integral elements of chronic inflammation are the structural and functional changes in extracellular matrix (ECM) compounds, including proteoglycans (PGs) and their sugar constituents—that is, glycosaminoglycans (GAGs) [8, 9]. The latter are negatively charged unbranched polysaccharides consisting of repeating disaccharide units of hexosamine and uronic acid or galactose. Chondroitin/dermatan sulfate (CS/DS), heparan sulfate/heparin (HS/H), keratan sulfate (KS), and hyaluronic acid (HA) represent the major species of GAGs. All GAG types, except HA, are covalently attached to the core protein, forming PGs, and exhibit various degrees of sulfation along the polysaccharide chain [10–12]. This structural heterogeneity allows GAGs to interact with and modify the actions of numerous cell-adhesion molecules, growth factors, cytokines, chemokines, components of ECM, proteases, and their inhibitors, which underlie their important biological functions, including cellular communication, cell signaling, and regulation of other biochemical pathways [10, 13]. Hence, every modification of GAG metabolism may play a crucial role in RA pathogenesis. The disturbed balance between biosynthesis and degradation of PGs/GAGs as a consequence of chronic inflammation during RA should be reflected in the concentration of plasma GAGs. Friman et al. [14] suggested that in patients suffering from an active, erosive form of RA, the total plasma GAG content is not different as compared to controls. On the other hand, Jura-Półtorak et al. [15] described an increase in the total plasma GAG level in patients with RA.

The introduction of biologic drugs which neutralize TNF-α activity was a breakthrough in RA treatment. The results of clinical trials revealed a significant reduction of disease activity and inhibition of radiological progression, as well as improvement in the quality of life and physical function in RA patients treated with TNF-α inhibitors (TNFαI) [16–18]. However, the effect of anti-TNF therapy on PG/GAG metabolism in RA is still unknown. Therefore, the main objective of this study was the quantitative evaluation of total plasma sulfated and nonsulfated GAGs in female RA patients, both before and during 15 months of anti-TNF-α therapy.

## Methods

### Patients and samples

Forty-five female patients (mean ± SD age 47.42 ± 13.70 years) meeting the 1987 revised criteria and the 2010 American College of Rheumatology (ACR)/European League Against Rheumatism (EULAR) diagnostic criteria for RA [19, 20] were recruited for this study. All subjects had a Disease Activity Score of 28 joints (DAS28) >  5.1 despite application of at least two disease-modifying anti-rheumatic drugs at trial entry. Exclusion criteria included previous treatment with biologic agents, withdrawing from the biologic therapy during the study period, presence of illnesses (other autoimmune diseases, infections, heart failure, diabetes mellitus, thyroid disorders, kidney, and liver disease or malignancies), pregnancy, and breast-feeding. Moreover, all of the female RA patients participated in Polish National Health Fund Therapeutic Programs employing TNF blockers—that is, B.33: “Treatment of aggressive forms of rheumatoid arthritis (RA) and juvenile idiopathic arthritis (JIA)” (03.0000.333.02), or B.45: “Treatment of an aggressive form of rheumatoid arthritis (03.0000.345.02)”—which were valid during 2012–2014. Twenty-two patients received adalimumab 40 mg subcutaneously every other week and 19 patients self-administered etanercept 50 mg by subcutaneous injection once a week, and four patients received certolizumab pegol 400 mg subcutaneously at weeks 0, 2, and 4 followed by 200 mg every 2 weeks thereafter for a period of 15 months. Patients were continuing current antirheumatic therapy, including methotrexate (25 mg/week) and prednisone (≤ 7.5 mg/day). All subjects were given folic acid in the dose of 5 mg/day. Concomitant medications remained unchanged for the duration of the study. Baseline characteristics of patients are presented in Table 1.

**Table 1:** Baseline characteristics of female RA patients treated with TNFαI

Characteristic	Value
All women with RA, *n* (%)	45 (100)
Age (years), mean (SD)	47.42 (13.70)
Disease duration (years), median (IQR)	7 (4–16)
BMI (kg/m^2^), mean (SD)	21.99 (2.26)
RF positive, *n* (%)	44 (97.78)
Anti-CCP positive, *n* (%)	45 (100)
SJC28, median (IQR)	8 (5–10)
TJC28, median (IQR)	14 (10–19)
VAS, median (IQR)	80 (80–80)
DAS28 ESR, mean (SD)	6.10 (0.58)
ESR (mm/h), median (IQR)	18.0 (11.0–33.0)
CRP (mg/l), median (IQR)	5.0 (4.0–14.9)
TNFαI therapy, *n* (%)	
Etanercept (Enbrel)	19 (42.22)
Adalimumab (Humira)	22 (48.89)
Certolizumab pegol (Cimzia)	4 (8.89)

*anti-CCP* anti-cyclic citrullinated peptide antibody, *BMI* body mass index, *CRP* C-reactive protein, *DAS28* Disease Activity Score based on evaluation of 28 joints, *ESR* erythrocyte sedimentation rate, *IQR* interquartile range, *RA* rheumatoid arthritis, *RF* rheumatoid factor, *SD* standard deviation, *SJC28* swollen joint count of 28 joints, *TJC28* tender joint count of 28 joints, *TNFαI* tumor necrosis factor-alpha inhibitors, *VAS* visual analog scale

The effectiveness of TNFαI treatment was assessed at the baseline of the study and 3, 9, and 15 months after starting anti-TNF-α therapy using the DAS28 indicator, calculated on the basis of the number of swollen and tender joints from among the 28 joints included, the erythrocyte sedimentation rate (ESR), and the patient’s global assessment of disease activity on a visual analog scale (VAS) of 100 mm. Furthermore, at each visit, patients were submitted to laboratory tests, such as complete blood count, markers of inflammation including the ESR and C-reactive protein (CRP), creatinine, and liver enzymes. Changes in clinical characteristics during the 15-month TNFαI therapy are summarized in Table 2. Patients who did not experience an adequate treatment response were excluded from the study. Adequate treatment response in accordance with the principles of the Polish National Health Fund Therapeutic Programs was defined as reduction in DAS28 > 1.2 after the first 3 months of therapy with a TNF-α inhibitor, and further reduction in DAS28 by 1.2 recorded during subsequent medical examinations performed 9 and 15 months after administration of the first dose of TNFαI.

**Table 2:** Time-course changes in biochemical, clinical, and functional measures during 15-month anti-TNF-α therapy

	Time after starting anti-TNF-α therapy			
	T_0_ (baseline)	T_1_ (3 months)	T_2_ (9 months)	T_3_ (15 months)
Women with RA, *n* (%)	29 (100)			
Age (years), mean (SD)	44.38 (14.17)			
Disease duration (years), median (IQR)	5 (3–8)			
BMI (kg/m^2^), mean (SD)	21.25 (2.28)			
RF positive, *n* (%)	29 (100)			
Anti-CCP positive, *n* (%)	29 (100)			
SJC28, median (IQR)	6 (5–10)	3 (2–3)^a, c, d^	0 (0–1)^a, b^	0 (0–0)^a, b^
TJC28, median (IQR)	14 (10–20)	5 (3–7)^a, c, d^	2 (1–2)^a, b, d^	0 (0–1)^a, b, c^
VAS, median (IQR)	80 (80–80)	50 (35–55)^a, c, d^	25 (10–30)^a, b, d^	10 (5–20)^a, b, c^
DAS28 ESR, mean (SD)	5.99 (0.50)	4.00 (0.73)^a, c, d^	2.74 (0.72)^a, b, d^	2.06 (0.64)^a, b, c^
Disease activity, *n* (%)				
High (> 5.1)	29 (100)	2 (6.90)	0	0
Moderate (> 3.2 and ≤ 5.1)	0	24 (82.76)	6 (20.69)	0
Low (≤ 3.2 and > 2.6)	0	3 (10.34)	12 (41.38)	6 (20.69)
Remission (≤ 2.6)	0	0	11 (37.93)	23 (79.31)
ESR (mm/h), median (IQR)	15.0 (10.0–31.0)	10.0 (8.0–17.0)	10.0 (8.0–14.0)^a^	11.0 (8.0–14.0)^a^
CRP (mg/l), median (IQR)	5.0 (4.0–9.2)	4.0 (2.0–4.0)	3.0 (1.30–4.0)^a^	2.0 (1.0–4.0)^a^
TNFαI therapy, *n* (%)				
Etanercept (Enbrel)	13 (44.83)			
Adalimumab (Humira)	14 (48.27)			
Certolizumab pegol (Cimzia)	2 (6.90)			

Differences noted for all variables (except DAS28 ESR) considered significant at *p* < 0.0083 by applying Bonferroni correction. Differences noted for DAS28 ESR considered significant at *p* < 0.001

*anti-CCP* anti-cyclic citrullinated peptide antibody, *BMI* body mass index, *CRP* C-reactive protein, *DAS28* Disease Activity Score based on evaluation of 28 joints, *ESR* erythrocyte sedimentation rate, *IQR* interquartile range, *RA* rheumatoid arthritis, *RF* rheumatoid factor, *SD* standard deviation, *SJC28* swollen joint count of 28 joints, *TJC28* tender joint count of 28 joints, *TNF-α* tumor necrosis factor alpha, *TNFαI* tumor necrosis factor-alpha inhibitors, *VAS* Visual analog scale

^a^Statistically significant differences compared to T_0_

^b^Statistically significant differences compared to T_1_

^c^Statistically significant differences compared to T_2_

^d^Statistically significant differences compared to T_3_

Twenty age-matched healthy female volunteers from the Medical University of Silesia in Katowice, Poland were investigated as controls. Subjects were selected after their medical history, clinical examination, and laboratory screening had been obtained. All volunteers enrolled in this study did not have any diseases that required hospitalization and did not undergo surgical procedures during the previous 3 years. Furthermore, the results of their routine laboratory tests (i.e., complete blood count, ESR, fasting glucose, fasting lipid profile, creatinine, liver enzymes, rheumatoid factor (RF), and CRP) were within the reference range. Subjects were excluded if they took steroidal or nonsteroidal anti-inflammatory drugs. None of the volunteers smoked cigarettes or had any history of drug or alcohol abuse. We selected women who could maintain a healthy body weight and had a body mass index (BMI) < 25 kg/m^2^.

On the day of collecting the plasma, prior to that procedure, patients met with rheumatologists for clinical visits, during which assessment of the patient, the physical visual analog scale of disease (VAS), the tender joint count of 28 joints (TJC28), the swollen joint count of 28 joints (SJC28), and the DAS28 were made. Venous blood samples were drawn between 7.00 and 9.00 am after overnight fasting, and were collected into citrate-treated (extraction and determination of plasma GAGs) and heparin-treated (measurement of plasma KS and HA levels) tubes. Plasma samples obtained from healthy subjects and RA patients were separated and stored at − 80 °C until the time of analysis.

During the entire investigation period we followed the guidelines and regulations of the Helsinki Declaration in 1975, as revised in 1983. The Ethical Committee of the Medical University of Silesia in Katowice approved the research protocol used in this study. All healthy volunteers and RA patients provided written informed consent.

### Extraction and determination of total plasma GAG levels

Sulfated GAGs were isolated using the methods of Lu et al. [21] and Capobianco et al. [22]. Firstly, plasma samples (1 ml) were pretreated for 24 h at 37 °C with Benzonase (E1014; Sigma) for the removal of nucleic acids. Next, plasma samples were submitted to exhaustive digestion with *Streptomyces griseus* protease (P0652; Sigma) in order to release GAG chains from plasma PG core proteins. This proteolysis was performed in 20 mM Tris–HCl, pH 9.0, containing 4 mM CaCl_2,_ at 56 °C for 24 h. After incubation, the samples were centrifuged at 19,000 × *g* for 30 min and the pellet was washed twice with the same buffer. The combined supernatants were applied to DEAE-Sephacel columns equilibrated with 0.1 M NaCl buffered with 20 mM Tris–HCl, pH 8.6, and columns were washed with the same buffer. GAGs were eluted with 2 M LiCl in 20 mM Tris–HCl, pH 8.6. Subsequently, GAGs were exhaustively dialyzed against water at 4 °C for 24 h and lyophilized before further analysis.

The total amount of GAGs was quantified as a hexuronic acid by the carbazole methods of Volpi et al. [23] and Filisetti-Cozzi and Carpita [24] as well as van den Hoogen et al. [25]. Then, 4.0 M ammonium sulfamate was added to each sample containing an aqueous solution of GAGs. The hydrolysis of GAGs to their monosaccharide constituents with simultaneous conversion of the glucuronic acid and/or iduronic acid residues to the corresponding furan derivative was carried out in concentrated sulfuric acid (95%) containing 0.025 M sodium tetraborate by heating the samples at 100 °C for 10 min. The contents of the tubes were then chilled in an ice bath and the background absorbance of the samples was measured at 525 nm with a microplate reader (Infinite M200; Tecan). In the next stage, furan derivatives present in the tested samples were coupled with 0.125% carbazole that was dissolved in absolute ethanol. The tubes were heated to 100 °C for 15 min and left to cool to ambient temperature. Afterward, the absorbance of the pink-colored samples was read again at 525 nm. For hexuronic acid quantification, a calibration curve was constructed using d-(+) glucuronolactone standard series (0–70 μg/ml). The background absorbance was subtracted from the second absorbance reading and the hexuronic acid concentrations were interpolated from the corresponding reference curve. The sensitivity of the reaction was approximately 1.5 μg for glucuronic acid. Testing of all samples was completed in 1 day, so interassay variation was insignificant. The intraassay variability of total GAGs was less than 3%.

### Measurement of KS and HA plasma levels

KS levels in plasma samples were measured in duplicate using an enzyme-linked immunosorbent assay (ELISA) from BlueGene Biotech (Shanghai, China) according to the manufacturer’s instructions. The minimal detectable KS level was 0.1 ng/ml. All samples were tested within 1 day, and thus interassay variation was insignificant. The intraassay variation of the KS levels was < 10%.

Plasma concentrations of HA were determined in duplicate using a TECO® Hyaluronic Acid Plus Test Kit provided by TECOmedical Group (Sissach, Switzerland), according to the manufacturer’s directions. This ELISA uses a highly specific hyaluronic acid binding protein (HABP) to capture HA and an enzyme-conjugated version of the HABP to detect and measure the HA captured from plasma samples. In brief, all plasma samples were diluted 50-fold with Sample Diluent. The analytical sensitivity was at 2.7 ng/ml. Testing of all samples was completed within 1 day to eliminate the effects of interassay variation. The intraassay coefficient of variation was < 2.9%.

### Statistical analysis

Data analyses were performed using STATISTICA version 12 (https://www.statsoft.pl). The normality of the distribution was verified using the Shapiro–Wilk test. Data not normally distributed were log-transformed prior to analyses. Variables are summarized as mean (SD) (for normal distribution) or median and interquartile (25th–75th percentile) range (for abnormal distribution). The homogeneity of variance was assessed using Levene’s test. Data were evaluated using a repeated-measures analysis of variance (RM-ANOVA) (normal distributed data) with a check for sphericity employing Mauchly’s test of sphericity, or using the RM-ANOVA Friedman’s test (nonnormal data). In the case of significant differences between subgroups, post-hoc analyses were based on the Tukey test (*p* < 0.05) or the Mann–Whitney *U* test (*p* value obtained after applying Bonferroni correction, *p* < 0.05; six possible comparisons).

## Results

### Clinical response

Out of a total of 45 female RA patients recruited for the study, 16 patients were excluded and the remaining 29 patients completed 15 months of treatment with TNFαI. In the excluded patients, TNFαI were discontinued due to the following reasons: no response in two patients, loss of response in three patients, intolerance in three patients, surgical procedures in four patients, and withdrawal of consent for participation in the therapy by four patients. Overall, 29 female RA patients who continued the TNFαI therapy for 15 months were included in our analysis and are presented in this study.

During the treatment with TNFαI, a significant clinical improvement in all RA patients was observed. Over the course of 3 months, 29 patients (100%) qualified as good responders in accordance with the EULAR response criteria [26]. What is more, this effect was sustained up to the 15th month. Remission of RA occurred in 38% of patients at the 9th month and in 80% at the 15th month of treatment, while the remaining patients experienced low activity. The DAS28 score was significantly reduced 3, 9, and 15 months after the initiation of TNFαI therapy when compared with baseline. Furthermore, CRP and ESR levels decreased significantly after 9 and 15 months of treatment (Table 2).

### Plasma levels of total GAGs, KS, and HA

The results regarding evaluation of plasma glycosaminoglycans (total GAGs, KS, and HA) were analyzed only in female RA patients who completed the whole 15-month TNFαI therapy (*n* = 29).

**Fig. 1:**
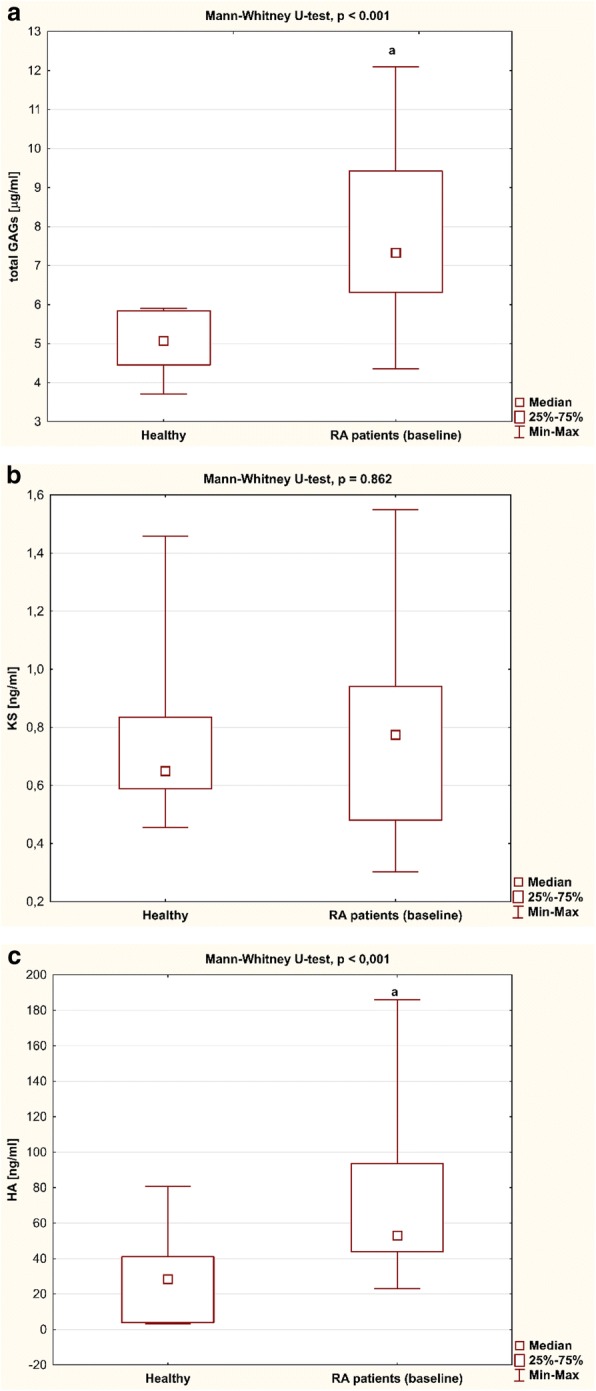
Plasma levels of (**a**) GAGs, (**b**) KS, and (**c**) HA in RA patients (*n* = 29) before anti-TNF-α therapy and in healthy subjects (*n* = 20). Data analyzed using Mann–Whitney *U* test. ^a^*p* < 0.001, compared to healthy subjects. GAG glycosaminoglycan, HA hyaluronic acid, KS keratan sulfate, Max maximum, Min minimum, RA rheumatoid arthritis

The concentrations of total plasma GAGs, KS, and HA in female RA patients before treatment with TNFαI and in healthy individuals are presented in Fig. 1a–c. Total GAGs and HA levels were significantly higher in RA women before anti-TNF-α therapy than in healthy subjects (both *p* < 0.001; Fig. 1a, c). KS levels were not different in RA women before biological treatment in comparison to those of the controls (*p* = 0.862; Fig. 1b).

**Fig. 2:**
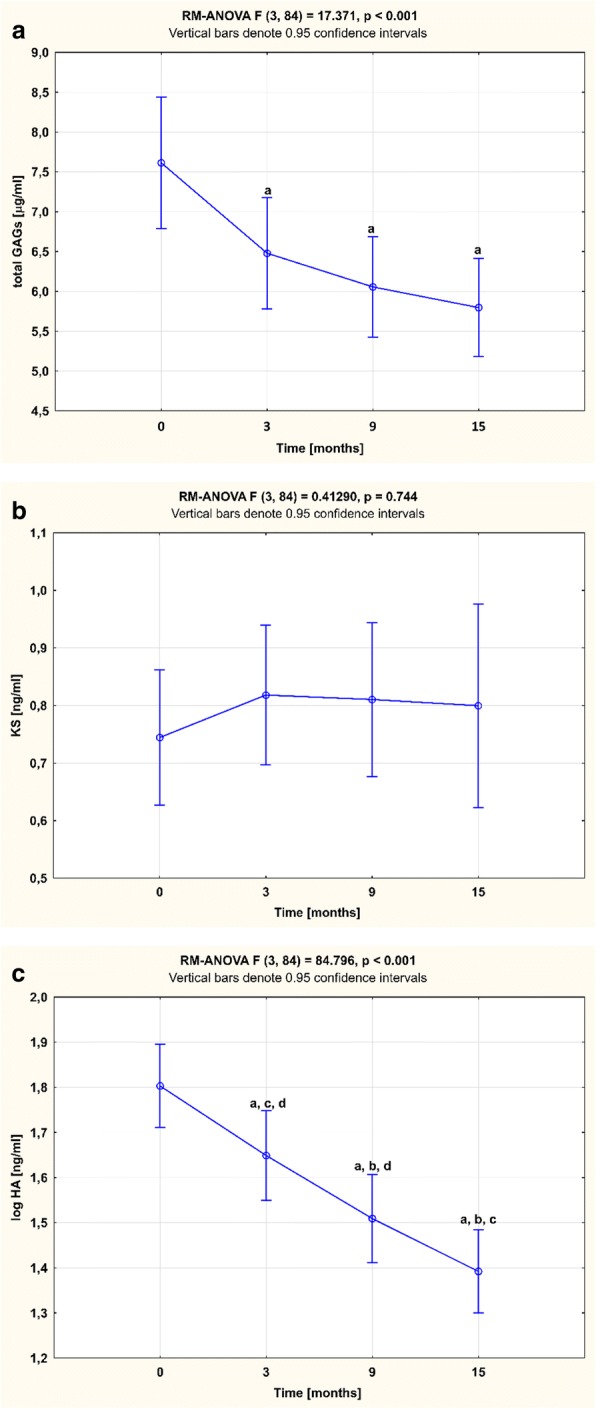
Temporal course of plasma (**a**) GAGs, (**b**) KS, and (**c**) HA levels in RA patients (*n* = 29) during 15-month anti-TNF-α therapy . Results expressed as mean (SD). Data analyzed using one-way RM-ANOVA, followed by Tukey’s multiple comparisons test. ^a^*p* < 0.001, compared to baseline (T_0_); ^b^*p* < 0.001, compared to 3 months after therapy (T_1_); ^c^*p* < 0.001, compared to 9 months after therapy (T_2_); ^d^*p* < 0.001, compared to 15 months after therapy (T_3_). GAG glycosaminoglycan, HA hyaluronic acid, KS keratan sulfate, RM-ANOVA repeated measures analysis of variance

**Fig. 3:**
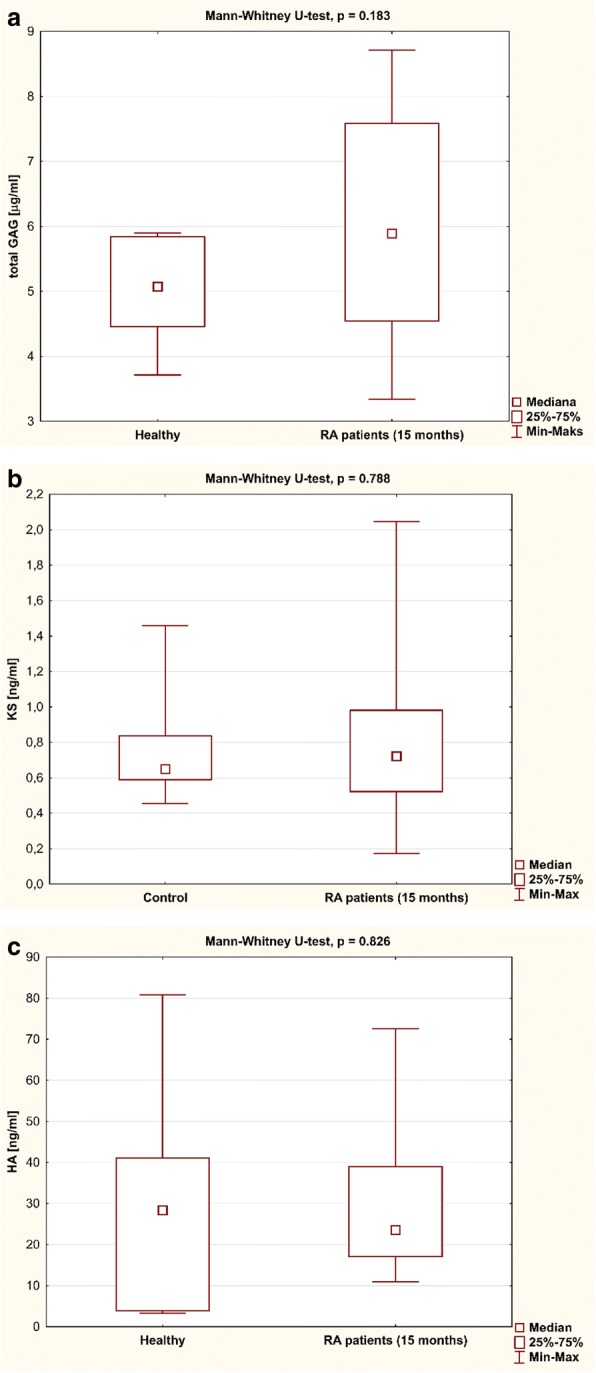
Plasma levels of (**a**) GAGs, (**b**) KS, and (**c**) HA in RA patients (*n* = 29) after anti-TNF-α therapy and in healthy subjects (*n* = 20). Data analyzed using Mann–Whitney *U* test. GAG glycosaminoglycan, HA hyaluronic acid, KS keratan sulfate, Max maximum, Min minimum, RA rheumatoid arthritis

Three months after the initiation of TNFαI therapy, a statistically significant decrease in total GAG levels was observed in RA patients (*p* < 0.001; Fig. 2a). Continued therapy resulted in a further decline (*p* < 0.001; Fig. 2a), reaching total GAG levels characteristic of the age-matched healthy controls after 15 months of treatment (*p* = 0.183; Fig. 3a). Similarly, HA levels decreased significantly in response to anti-TNF-α therapy (*p* < 0.001; Fig. 2c). Furthermore, anti-TNF-α treatment also led to normalization of HA in RA patients (*p* = 0.826; Fig. 3c). In contrast, KS levels in RA women were not affected by the treatment (*p* = 0.744; Fig. 2b) and were not significantly different from those in healthy subjects after 15 months of TNFαI therapy (*p* = 0.788; Fig. 3b).

## Discussion

TNF-α inhibitors provide a new standard in the treatment of RA. TNFαI administration for 15 months resulted in improvement in terms of disease activity, as shown by the decrease of DAS28 and CRP and the achievement of full remission or low disease activity in all RA patients. We also showed that this beneficial effect was associated with improvement in metabolism of ECM components, assessed through plasma sulfated and nonsulfated GAG levels in female RA patients. Indeed, a decrease in the total GAGs as well as HA levels toward normal values was observed under anti-TNF-α treatment. Only the plasma concentration of KS in RA women was not affected by the treatment. To the best of our knowledge, this is the first study that reports an association between good clinical response to anti-TNF treatments and the plasma GAGs in RA patients. The quantitative changes in the level of plasma GAGs observed in female RA patients during biological therapy seem to result from an effective inhibition of the key proinflammatory cytokine, TNF-α, rather than from direct impact of TNFαI on the tissue ECM remodeling. This suggestion is supported by the results of Jura-Półtorak et al. [15], who evaluated the plasma GAG profile in relation to disease activity in RA patients treated with conventional synthetic disease-modifying anti-rheumatic drugs (i.e., methotrexate or sulfasalazine). Similarly to our results, they demonstrated a significant increase in the total plasma GAG level in RA patients with a high disease activity (DAS28 >  5.1) in comparison to patients with a low disease activity (DAS28 ≤ 3.2) [15]. Thus, the primary determinant of tissue PG/GAG turnover in RA patients is not the type of medication used for treatment of RA, but the control of disease activity. Moreover, consistent with our results, Jura-Półtorak et al. [15] found that the plasma level of GAGs was significantly elevated in RA patients with a high disease activity in comparison to healthy individuals. On the contrary, Friman et al. [14] showed that the plasma GAG concentrations in female patients with an active, erosive form of RA and in healthy subjects are similar. These discrepancies can result from methodological differences, especially with respect to the binding properties of a bed used in the ion exchange chromatography, that are crucial for the isolation of the plasma GAGs, which are characterized by huge heterogeneity in their electrical charge. In addition, the aforementioned divergences between our results and those of Friman et al. [14] might also be caused by differences in the disease activity of female RA patients, disease duration, as well as a type of anti-rheumatic drugs used. Summarizing, the blood accumulation of the total GAGs in the course of RA observed by us may indicate an increased tissue ECM turnover that is dependent on the disease activity.

Considering our results, we can suppose that decreased levels of total plasma GAGs during 15 months of TNFαI treatment might be due to reduced enzymatic and nonenzymatic catabolic processes. Matrix metalloproteinases (MMPs) and the family of proteins termed a disintegrin and metalloproteinase with a thrombospondin motifs (ADAMTS), as well as reactive oxygen species (ROS), participate in the turnover of tissue PGs/GAGs [9, 27–29]. With regard to the enzymes discussed earlier, the levels of MMP-3 and MMP-1 in serum of RA patients were found to be downregulated by TNFαI therapy [30–33]. Little is known about the effect of TNF-α inhibitors on ADAMTS activity in RA patients. However, inhibition of radiographic progression by TNFαI has been extensively documented through many clinical trials and it can be assumed that this beneficial result is at least partially related to the decreased activity of aggrecanase-1 (ADAMTS-4) and aggrecanase-2 (ADAMTS-5), proteinases responsible for cleavage of the main cartilage matrix PG, aggrecan [28, 34]. Another mechanism of alterations to the plasma GAG level in RA women may be connected with the ability of TNFαI to promote programmed cell death through the inhibition of nuclear factor-κB (NF-κB) activation. As reported previously, etanercept as well as infliximab induce apoptosis of synovial fluid monocytes/macrophages, which are a constant source of MMPs and ADAMTS [28, 35]. Furthermore, TNF-α blockade normalizes the number of circulating monocytes in RA patients [36].

Besides the proteolytic breakdown of the ECM, oxidative damage plays a very prominent role in postsynthetic modifications of matrix components [29]. It is well known that excessive ROS formation in RA patients leads to peroxidation of the core proteins of PGs as well as the partial cleavage of GAG chains, thereby increasing the plasma GAG content [29, 37]. Thus, it seems that normalization of the total plasma GAG level in RA women found in our study should be partly a reflection of the decreased nonenzymatic free radical degradation of these glycans. Indeed, anti-TNF-α therapy has been shown to suppress ROS generation in RA patients. It is reported that TNF-α blocking drugs reduce serum levels of reactive oxygen metabolites (ROM) as well as serum or urine levels of oxygen stress markers, including pentosidine, 8-hydroxy-2′-dexoyguanosine (8-OHdG), and *N*^ε^-hexanoyl lysine (N^ε^-HEL) [38–40]. Inhibition of neutrophil migration into inflamed joints after initiation of anti-TNF-α treatment in RA patients has also been described [41]. The mentioned cells are abundant in RA synovial fluid, and their release of oxygen-derived free radicals and other inflammatory mediators may intensify the catabolism of PGs/GAGs [41].

Because most GAG types are also found in noncartilaginous tissues in significant amounts, their plasma levels do not essentially reflect cartilage PG catabolic activity in RA patients [42]. Keratan sulfate is an exception, in that more than 95% of KS is found in the aggrecan, a large aggregating PG of hyaline, elastic, and fibrous cartilages [42]. Thus, this may indicate that most plasma KS should come from cartilage PG degradation. Elevated levels of plasma KS have been observed in patients with osteoarthritis, as well as in healthy individuals characterized by higher sports activity [43, 44]. In previous studies, the plasma concentrations of KS in RA patients have been shown to increase or decrease [15, 45–47]. In the present study, no significant difference was found in plasma KS levels between female RA patients and controls. We also showed that KS levels in RA women remained unchanged during 15 months of anti-TNF-α therapy. Our findings differ from those of Niki et al. [30], who demonstrated that the infliximab therapy-triggered increase of serum KS levels was more significant in the established RA patients than in the group of early RA patients. The possible explanation for these discrepancies could be connected with methodological differences in measurement of KS. In addition, these divergences might be caused in part by differences in gender of the patients, in the disease activity, and in amount of habitual exercise. In summary, the data presented here suggest that the potential usefulness of plasma antigen KS as an indicator of altered metabolic processes occurring in cartilaginous structures of RA patients during TNFαI therapy needs to be reevaluated.

In contrast to KS, plasma levels of the nonsulfated GAG hyaluronan could be helpful in predicting the efficacy of anti-TNF-α therapy in RA. The circulating levels of HA were greater in female RA patients before anti-TNF-α treatment when compared with age-matched healthy individuals. Similar findings have been reported in previous studies, none of which determined the gender of the patients [15, 48, 49]. In addition, TNFαI therapy led to a significant decrease in HA level, down to the values observed in healthy subjects. The outcomes of this study correspond with the results of Niki et al. [30]. They demonstrated that in the early RA group the serum level of HA gradually decreased during the 54-week infliximab therapy. Moreover, Niki et al. [30] found a strong linear correlation between the blood HA level in patients with early RA and DAS28, as well as inflammatory markers, such as CRP. These results may indicate that HA is an active participant in the inflammatory process accompanying RA. In the systemic circulation and in the synovial fluid of RA patients, the presence of low molecular weight fragments of hyaluronan (LMW-HA) was revealed. Such LMW-HA resulting from tissue HA depolymerization elicit proinflammatory responses by modulating toll-like receptor-4 or by activating NF-κB. NF-κB allows a vicious circle of chronic inflammation in RA by stimulating the expression of several proinflammatory cytokines such as interleukin (IL)-1, IL-6, and TNF-α, which in turn induce alterations in HA metabolism [8, 9, 12, 29, 50]. Since hyaluronan is the most susceptible among all of the GAG types to degradation in the presence of ROS, it may be assumed that suppression of free radical-mediated HA fragmentation may be the main cause of normalization of plasma HA in the course of biological therapy that was observed in our study.

## Conclusions

In summary, the results of our study have shown for the first time that anti-TNF-α therapy, which contributes to a clinical improvement in female RA patients, also has a beneficial effect on metabolism of tissue PGs/GAGs. Normalization of plasma levels of total GAGs and HA in RA patients suggests improvement of the ECM remodeling balance, mainly due to a decrease in PG/GAG breakdown. Our observations provide an additional mechanism for explaining cartilage-protective effects associated with anti-TNF-α treatment.

## Abbreviations

8-OHdG: 8-Hydroxy-2′-dexoyguanosine
ACR: American College of Rheumatology
ADAMTS: Disintegrin and metalloproteinase with a thrombospondin motifs
BMI: Body mass index
CRP: C-reactive protein
CS/DS: Chondroitin/dermatan sulfate
DAS28: Disease Activity Score based on the evaluation of 28 joints
ECM: Extracellular matrix
ELISA: Enzyme-linked immunosorbent assay
ESR: Erythrocyte sedimentation rate
EULAR: European League Against Rheumatism
GAG: Glycosaminoglycan
HA: Hyaluronic acid
HABP: Hyaluronic acid binding protein
HS/H: Heparan sulfate/heparin
IL: Interleukin
KS: Keratan sulfate
LMW-HA: Low molecular weight fragments of hyaluronan
MMP: Matrix metalloproteinase
NF-κB: nuclear factor-κB
N^ε^-HEL: *N*^ε^-hexanoyl lysine
PG: Proteoglycan
RA: Rheumatoid arthritis
RF: Rheumatoid factor
RM-ANOVA: Repeated-measures analysis of variance
ROM: Reactive oxygen metabolites
ROS: Reactive oxygen species
SJC28: Swollen joint count of 28 joints
TJC28: Tender joint count of 28 joints
TNF-α: Tumor necrosis factor alpha
TNFαI: Tumor necrosis factor-alpha inhibitors
VAS: Visual analog scale
